# Ad hoc interpreters in South African psychiatric services: service provider perspectives

**DOI:** 10.1080/16549716.2019.1684072

**Published:** 2020-01-14

**Authors:** Sybrand Hagan, Xanthe Hunt, Sanja Kilian, Bonginkosi Chiliza, Leslie Swartz

**Affiliations:** a Department of Psychology, Stellenbosch University, Stellenbosch, South Africa; b Department of Psychiatry, Stellenbosch University, Tygerberg Hospital, Stellenbosch, South Africa; c Department of Psychiatry, University of KwaZulu Natal, King Edward Hospital, Durban, South Africa; d Alan J Flisher Centre for Public Mental Health, Cape Town, South Africa

**Keywords:** Interpreting, cultural broker, language, psychiatry, mental health, Limited English Proficiency

## Abstract

**Background**: Language and communication lie at the heart of good quality mental health care and are a vital, yet complex, part of the diagnostic and treatment process. In South African mental health care, ad hoc interpreting arrangements are the status quo. However, these can cause both clinician and patient shame and distress.

**Objective**: Though this issue has been researched from the point of view of informal interpreters, relatively little is known about psychiatrists’ experiences of working with ad hoc interpreters. This study is part of an attempt to bridge that gap.

**Methods**: We made use of a cross-sectional qualitative interview design. We interviewed seven psychiatrists working at a psychiatric hospital in the Western Cape of South Africa. Data were analysed manually using thematic analysis.

**Results**:Five main themes can be identified from the data: miscommunication and other difficulties associated with the language barrier; the language barrier between clinicians and patients and a need for language services; participants’ views on interpreting; the role of age, culture and gender; and the use of trained and untrained interpreters and using staff as interpreters.

**Conclusions**: Psychiatrists experience numerous difficulties in conducting their work due to the language barrier. This has an impact on their ability to provide adequate mental health care to patients. There is a need for better language services to ensure that everyone that seeks mental health care can receive the same level of care. Currently, one’s ability to speak a certain language will have a significant impact on the quality of care received.

## Background

Language and communication lie at the heart of good quality mental health care and are a vital, yet complex, part of the diagnostic and treatment process [1–4]. Words and the relationship between clinician and patient are two of the main components of mental health care [5]. It is of central importance that mental health workers and patients understand what each other are saying [4,6]. Good quality health care relies on effective communication.

Communication generally happens through the medium of language. This enables patients to report symptoms to clinicians [4,7]. Language barriers pose a significant threat to delivering good quality health care, but also to the access of treatment, patient satisfaction, clinician-patient relationship, treatment adherence and health outcomes [8,9].

### Communication and health care quality

The quality of communication is a useful proxy for the assessment of the quality of care [10]. Patients tend to filter out important information when they are forced to use a language that is not their first language [7]. Successful communication goes beyond conversational competence between the different people involved. There are particular contextualised tasks which need to be undertaken in the clinical encounter [6].

Without a common language, clinicians are mostly unable to communicate effectively with their patients, and are thus unable to assist their patients properly [9,11]. If patients cannot be understood, they cannot receive adequate health care [3], leading to inadequate or improper care [12]. Better patient knowledge leads to a better prognosis, which makes it important for patients to understand the purpose and nature of the health care that they are receiving [2]. There is thus a link between effective communication and appropriate health care [11,13]. Not only does the language barrier negatively affect the care of the patients, but clinicians and other health care workers feel defeated, helpless and vulnerable [14].

### Communication and the therapeutic relationship

To ensure the provision of good quality health care, it is of vital importance that there is a good therapeutic relationship between clinician and patient, which is difficult to manage if successful communication cannot take place [15]. The therapeutic relationship relies heavily on the clinician’s ability to form a trusting and honest relationship with a patient, which is near impossible if they do not share a common language [16,17]. Miscommunication may be felt by clinicians as a loss of therapeutic momentum [18], which may result in clinicians feeling detached from the therapeutic relationship [19].

Priebe and Mccabe [20] state that there is evidence to suggest that the therapeutic relationship predicts the outcome across various psychiatric settings. Thus, ineffective communication can lead to ineffective therapy.

### Culture and cultural nuances

Mental health services rely heavily on the communication of complex ideas and feelings [21]. Language, the major medium of this communication, is a powerful transmitter of culture [11]. Difficulties around language access are often accompanied by cultural differences, which lead to further misunderstandings [17]. Health care institutions and clinicians have the difficult task of delivering culturally competent and culturally responsive care [22], which is seriously threatened by the language barrier. The language barrier is of course part of the issue of cultural and ethnic diversity [22].

The meaning of cultural nuances and words, especially when denoting emotional states, cause difficulty when translated and interpreted, as meanings are not always equivalent across languages and cultures [11,23]. The meanings of words can change when translated. Even when patients understand a language to some extent, this does not mean that they will be able to effectively communicate in that language [11]. This is especially true in the complex world of mental health care, as clinicians rely on patients to understand a diagnosis and/or treatment options. Despite the growing number of Limited English Proficiency (LEP) patients, the effect that this has had on health care outcomes has received little research attention [24].

### Dominant language

Users of language are often unaware that through the power of language, they create meanings, beliefs and attitudes [1]. English is regarded as the dominant language of health care in Anglophone countries [25]. As a result of this dominance, people with LEP may not receive optimal health care [26]. Karliner et al. [25, p. 728] define individuals with LEP as not being ‘able to speak, read, write, or understand the English language at a level that permits them to interact effectively’. The delivery of optimal health care to LEP patients is adding an extra challenge to mental health care [27]. Access to good quality health care should be available to all, and not just to those fluent in the dominant language [28,29]. Various consequences and difficulties for patients and clinicians are created due to the language barrier [30–32], with linguistic and cultural communication difficulties cited as two of the most common barriers to health care access [2,33].

### The South African context

In South African mental health care, ad hoc interpreting arrangements are the status quo. However, these can cause both clinician and patient shame and distress [3]. During the Apartheid years in South Africa, mental health professionals were required to speak only Afrikaans and English [6]. This meant that very few mental health professionals could speak any of South Africa’s indigenous languages, apart from Afrikaans [6]. The advent of democracy in South Africa has brought many benefits, but for the vast majority of people that access public health care there has been no substantial change [3]. Very few mental health care professionals speak an indigenous African language, with Afrikaans and English remaining the dominant languages, and little systematic attention to ethnic differences [6,34–36].

There are major problems in delivering good quality health care to all South Africans, as hospitals are ill-equipped to deal with the language barrier [37,38]. Using the Western Cape as an example, Afrikaans and English still enjoy the highest status, even though Xhosa is also an official language [37,39,40]. This creates a major barrier to health care, as these cultural and linguistic barriers limit equal access to mental health care services [41]. Language barriers are commonplace in clinical work in South Africa, so much so that they have become part of everyday life [35,42].

### Rationale for the present study

Language has an immense impact on the success of psychiatric assessment and diagnosis. There is also a strong link between culture and language use [12]. Successful linguistic and cultural communication between patient and clinician is essential to ensure good quality health care.

Communication across language barriers has received much attention in countries like the United States, however, in low- and middle-income countries like South Africa, communication tends to have a low priority due to other pressing issues in the health system [43].

In South Africa, clinicians are commonly forced to make use of ad hoc arrangements to communicate with patients [3]. These arrangements are clearly not ideal, as can be seen in studies by Hagan et al. [26], Hagan [44], Kilian [45], Kilian et al. [39] and Steyn [46]. Though this issue has been researched from the point of view of informal interpreters, relatively little is known about psychiatrists’ experiences of working with ad hoc interpreters. This study is part of an attempt to bridge that gap.

The study we report on here forms part of a larger project examining language issues in mental health care in our context. By way of background for this this study, it is important to point out, as we have argued in our previous work, that the question of language in mental health care, especially in a divided society interleaves with broader concerns about patient rights, about the complicity of practitioners in reproducing care which is not optimal. One example of this problem is that there is currently no standard of care for work through and interpreter in the South African context [3]. It is also important to consider the ways in which the practice of psychiatry in a postcolonial context needs to be understood in terms of global economies of wealth and power [3,4,47]. This said, it is important if change is to occur to understand the everyday context of the work from the position of those doing the work [48].

## Methods

### Research design

We made use of a cross-sectional qualitative interview design. Ethical approval for this study, which is part of a larger study on language issues/interpreting in mental health care in Cape Town, was obtained from the respective Departmental Ethics Screening Committee (DESC), the Humanities Research Ethics Committee: Humanities (REC), Stellenbosch University and from the respective hospital committees. All participants completed informed consent forms, and their participation was voluntary.

### Context

Data were collected at one of the three major public psychiatric institutions in the Western Cape Province, South Africa. The institution is affiliated with a leading research university which trains psychiatrists. Most patients seen by the trainees come from impoverished communities in the Cape Town area.

### Participants

Participants were selected according to criteria of relevance to the research aims and objectives (purposive sampling) [49]. The sample consisted of seven psychiatrists. Five of the participants were female and two were male. Five of the participants were first language Afrikaans, and two first language English. Participants were chosen on the basis that they are psychiatrists at a mental health hospital in the Western Cape.

### Data collection

Data were collected by way of semi-structured interviews. Interviews took place at the participants’ place of work and were conducted by the first author.

### Data analysis

The interviews were audio-taped and transcribed. Thematic analysis was used to analyse the transcribed data. The first step in this process entailed reading and rereading the transcribed text, generating initial themes [50]. Themes were then clustered into related ideas, defined and named [50]. Quotes from the data were then used to capture the essence of each theme. Themes were developed into a more in-depth analysis by writing and rewriting, which also helped in examining the relationship between themes [50]. Lastly, the relationship between themes and the broader socio-cultural context was examined [50].

## Results

Examples of verbatim extracts from the interviews are included to illustrate and substantiate research findings. These extracts are presented with pseudonyms to protect the privacy and confidentiality of participants and institutions. Five main themes can be identified from the data: miscommunication and other difficulties associated with the language barrier; the language barrier between clinicians and patients and a need for language services; participants’ views on interpreting; the role of age, culture and gender; and the use of trained and untrained interpreters and using staff as interpreters.

### Miscommunication and other difficulties associated with the language barrier

There are numerous difficulties when there is a language barrier between clinician and patients.

When there is a language barrier a lot of important information can get lost in the interaction; sometimes clinicians might not even be aware that information is missed. This is expressed in the following quotes by participants:

***P1***: I just realised again, how much gets lost in translation.

***P1***: I think in a language that you don’t understand at all, you’re not even going to realise that it gets lost.

Several participants noted that information still appeared to get mislaid when an interpreter was present. As one participant reported, the interpreter and patient might have a long conversation, but the interpreter only communicates a short phrase back to the clinician:

***P3***: Yes, when you work with an interpreter, it depends on who your interpreter is. Because, the patient will give you three hundred words, and then the interpreter turns to you and says, ‘Yes, he says it’s true’.

Another challenge when working with an interpreter was that they could make their own interpretation of what the patient is saying:

***P4***: But what happens often, the interpreter is busy to give their own interpretation of what the patient said.

A more practical example of this is when a patient is seemingly talking what a participant calls ‘nonsense’. Interpreters would then try to make sense of what the patient is saying, instead of repeating the words verbatim:

***P5***: So someone would say a thing like the patient was speaking nonsense or they would just, they would literally interpret what the patient had said in their own way, trying to make sense instead of repeating verbatim what was being said.

One participant reported that even when a patient is able to communicate in a certain language, they tend to revert back to their first language if they are acutely psychotic or manic:

***P2***: Uhm, the problem is that when people are acutely psychotic or manic, they tend to revert back to their first language and then it’s more difficult to communicate with them. Uhm, and then in those circumstances it certainly becomes more important to be able to communicate to that person using their first language.

Interpreters also tend to use the wrong terminology, thus not repeating exactly what the clinician is trying to communicate. It is, however, important to remember that interpreters that are used are not necessarily clinically trained and might not know the correct terminology. Moreover, participants noted that, not only does the actual content of a conversation get lost, but it is also difficult to pick up on the emotions behind what is being said:

***P7***: Because we can also observe the patient’s emotional response to the content and evaluate it, and you lose that as soon as you use an interpreter.

Due to a language barrier, coming to the correct diagnosis can also be difficult, and clinicians might completely miss a diagnosis. As one participant noted:

***P5***: You know, you can completely miss a diagnosis unless you’re aware of the impact of culture, language and all those things we’ve been talking about.

One participant also stated that interpreters may ‘clean up’ what the patients are saying. By doing this, they might be unconsciously hiding symptoms that the patient has:

***P7***: You know, I think sometimes the interpreter tries to clean up the content for you, and they don’t realise that they are actually censoring the psychotic nuances, the illness aspects of the language.

The majority if the participants reported that it was difficult to build a therapeutic relationship with patients when there is a language barrier. As one participant noted:

***P1***: You cannot build an alliance. You cannot at all.

***P1***: And that attachment is difficult through a third person, you can get information and do an evaluation, but it is difficult to build a therapeutic relationship, a warm therapeutic alliance.

When making use of interpreters, participants also noted that ethical issues arise, most importantly, ensuring confidentiality becomes difficult:

***P7***: I have, in the past there was a security guard at a ward that I asked, which I know was asked often to interpret, and I have explained confidentiality issue to her. But I’m not, I don’t always feel comfortable to ask security, because they don’t have the same level of understanding about confidentiality as other staff members.

These informal interpreters are also not necessarily trained to communicate with patients and may act inappropriately. Especially when an interpreter is inexperience, they might not know how to act in the presence of patients:

***P2***: Uhm, to remind that person or to know what it is you need to inform the interpreter of. For example, the need of confidentiality, the need for treating the patient with respect at all times. And I think that’s, I’ve certainly seen, uhm, inexperienced interpreters sometimes being inappropriate in terms of laughing at someone or becoming annoyed with someone because they’re not answering a question appropriately. Uhm, it’s a very delicate space I think.

The language barrier and use of interpreters also put strain on an already busy and full schedule, as spending time with patients that do not speak the language of a clinician often leads to a longer interview. As one participant explained:

***P5***: It’s just that often, you know, it’s just, you know, busy clinical situation and somebody comes in and, uhm, you may not necessarily, you know, may not necessarily have the time to, to use the interpreter adequately, if that make sense?

Finally, one participant reported that when an interpreter is used, the clinician has to ask their questions and then wait for the answer, which can be an irritating process:

***P7***: So it is, it’s actually a very irritating type of interview, because you ask a question, then you wait, and then when the answer comes back you have to … You see, the answer is not elicited in the same way as if you asked it directly. So the interview takes twice as a long.

### The language barrier between clinicians and patients, and a need for language services

All seven participants spoke and understood English and Afrikaans, but none were fluent in any of the other nine official languages of South Africa. Some participants had a rudimentary understanding of isiXhosa but were not fluent enough to conduct an interview. As one participant explained:

***P1***: In isiXhosa I will only be able to do a physical examination, like look here and lift this and put out your tongue. But my psychiatric interview I will not be able to do, only in Afrikaans and English.

The hospital where the current research was conducted falls in a catchment area where there are mostly Afrikaans and English patients, although there are some isiXhosa patients. In instances where a patient is not able to communicate in English or Afrikaans, it is often the responsibility of the clinician to be creative, and sometimes they rely on their being a staff member on duty that can speak the language:

***P1***: Uhm, we have, we try to be as creative as possible. You know, at one stage in our alcohol ward we tried to organise an interpreter and run a specifically isiXhosa group. Uhm, the problem is that they then stagnate, in other words, if they know that group is for example once a quarter, then they have to wait a full quarter to have access to that service, which isn’t necessary sensible. And we also found that it was difficult to get referrals. Because it is not a general service, but at the moment if we have patients that struggle with isiXhosa, then we have an isiXhosa speaking nurse. We then have to hope that she is on duty most of the time to help if extra explanation is necessary.

A participant noted that they would even consider asking a colleague from a different department/ward to help with interpreting. One participant explained:

***P7***: And then I will sometimes ask my outpatients people to interpret for me. Especially what happens in accidents, you are sitting with the patient right now. You can’t let the patient come back tomorrow and organise an interpreter for tomorrow. You want to know what the problem is now. So then you have to make a plan. And then sometimes, sometimes I will ask staff members.

In terms of therapy groups, some are only conducted in English and Afrikaans, and patients that speak a different language need to either wait until an interpreter is available to join a group, or patients are simply not able to join groups:

***P1***: Uhm, sometimes there is a patient, because the programme is presented in English and Afrikaans, it is required that they speak these languages, but sometimes there is a patient that is not very good, who is isiXhosa speaking. But the majority of the patients are Afrikaans, well almost everyone can speak Afrikaans and English well enough to participate.

Regarding treatment, when no one was available to interpret, one participant reported that clinicians would simply diagnose based on the clinical picture, taking a near educated guess regarding medication and dose. One participant noted:

***P4***: Uhm, we’ve had one or two patients where nobody, there’s no, nobody speaks English, and sometimes we try to work through their own consultants, but if they are not there, then we have to go on clinical image. So this, the patient looks like this, you know, how much Lorazepam he needs, stuff like that.

One participant also explained that with certain disorders, language becomes an issue even if the patient can communicate in a second language, as they might revert back to their first language when distressed:

***P2***: Uhm, the problem is that when people are acutely psychotic or manic, they tend to revert back to their first language and then it’s more difficult to communicate with them. Uhm, and then in those circumstances it certainly becomes more important to be able to communicate to that person using their first language.

When patients are forced to communicate in a second or even third language, communication might be able to take place, but the finer nuances of the interview are lost. Patients might feel less comfortable in a second or third language, and as one participant stated, this might result in patients ‘hiding’ information or symptoms, as they might be more guarded:

***P4***: When they speak in their second or third language, then, ugh, I don’t understand all of the physiology behind it, but then, then they can in a way, they are more guarded and they can hide it more easily, because they have to translate everything in their heads.

Due to the catchment area of the particular hospital, the need for language services was not expressed as being obvious amongst participants, however there was still agreement that there is a need to better serve patients who speak languages other than English and Afrikaans:

***P7***: I think the problem was that we definitely have a need, but that it is not a consistent need. You know, you don’t need an interpreter every day for a full day.

One participant reported that if the programme that they run was offered in more languages, there would likely have been more patients that speak indigenous African languages:

***P1***: I think there is a need, a big need. Maybe not at this point in my services, but wider I think there definitely is a need, and I think if we offered a more language competent programme, we would have more than likely had more patients that would have wanted the service.

Although this would be very difficult to achieve, one participant noted that in a country like South Africa, clinicians should always try and speak to a patient in their first language:

***P2***: So I think that in a country like South Africa, when you are seeing someone, it’s important to try and speak to them in their first language. It’s a sign of respect and it puts them at ease and it builds rapport. So you’re doing it to consider culture and language preference in that regard.

### Participants’ views on interpreting

The majority of participants reported that if used correctly – with adequate time for scheduling, and trainings – interpreters could be an effective and useful resource. It was also beneficial if the same interpreter could be used over a lengthy period of time. This, however, is not a luxury that is always available, and it can be difficult to access people to interpret:

***P6***: And I still have patients that I see, because we have been using the same woman for years. Uhm, and so … If I, in terms of, I would guess eighty percent of such interviews that I have ever done, was research related, interviews where I, which I have done a lot.

The presence of a third party in the room could also be uncomfortable for already vulnerable patients:

***P2***: Of course, and I’m thinking more now in terms of the therapeutic session. Uhm, of course, because you have someone that’s in a very vulnerable, fragile space. Uhm, and a lot of clients struggle with the idea of making themselves even more vulnerable to someone, uhm, exposing themselves to a complete stranger. To then expose yourself to two people who you may feel are working with each other and will build opinions of you together, uhm, that’s, that’s difficult. And I think that anyone would prefer not to have to experience that.

Although having an interpreter present could have benefits, it also incurs other challenges, like confidentiality, and questions arise, such as whether the interpreter will be empathic. As a participant explained, interpreters that are used have not always received adequate training:

***P2***: So I think that there’s a need to be able to communicate effectively, not only with your patient, but with your interpreter, and the need for the interpreter to be empathic and retain confidentiality at all times. You’re expecting a lot out of someone who probably has not had adequate training.

Participants reported that there were interpreters at the hospital for a while, but this service has since stopped. When this service was available, it was sometimes difficult to make appointments with the interpreter, as they would often be busy somewhere else, and as a participant noted, there wasn’t always time to wait for an appointment with an interpreter:

***P3***: No, just saw them. The thing is, for an interpreter to work, they have to be available when you need them. But now it works like this, this interpreter is apparently somewhere on the grounds. I hope she’s still on the grounds. So now you have to make an appointment. There’s no time for appointments. I am sitting with eight patients at the clinic. I can’t wait for an appointment. It just doesn’t work.

During the time when interpreters were employed at the hospital, it was possible to run groups in isiXhosa. Unfortunately, this was a limited resource, and these dedicated interpreters were not always available. One participant explained, however, that some of these interpreters are still working at the hospital but have taken a more administrative role.

### The role of age, culture and gender

The impact of age, culture and gender on mental health interviews is potentially profound. Considering that when there is also a language barrier and the potential of a third presence in the room, this becomes even more complicated. As can be seen below, participants reported that the age of a patient has the most impact when they are interviewing an older patient:

***P5***: Ja, I do, I think [age] does impact. I think, you know, it’s difficult, you know, for someone who’s lived a lifetime and then somebody much younger comes in and has an opinion. I think that can be difficult.

The impact of age could also be linked to culture, as in certain cultures it is expected that older patients be addressed in a certain way:

***P7***: And I think with older people, there is obviously more respectful ways of, you know. For example, for people that are a lot, especially elderly patients, I will often address them as sir/ma’am. I definitely adjust my interview style with patients. Not only because of respect, but also to offer them the opportunity to relate on a deeper level, and it enhances the therapeutic relationship. So I think you adjust to the situation, and I think there are also cultural aspects that also play a role.

One participant also noted that older patients who are first language isiXhosa speakers might have a lower level of education. As a result, these patients might be less able to communicate in English than the younger generation. Often when there is a language barrier, patient and clinician are also from different cultures. This means that clinicians are often faced with cultural barriers as well. One participant explained that certain cultures might communicate in quite a symbolic way, which can be difficult for clinicians to interpret if they do not understand the culture:

***P1***: It is frustrating, and I think what was interesting for me with the interpreter, was that they had to, uhm, the Venda’s speak very symbolically, so the nurse also had to interpret the symbolism. So the patient will come, and they will say the problem is a snake bite, then you search for it and then there is no snake bite … and then the snake bite is symbolic of a pain that is actually an emotional problem, the husband has an affair. So she is speaking about a snake bite, but actually her husband is having an affair and that is actually the problem, and she is upset about this. So that symbolism, what certain things mean, the nurses had to help with that, because our thinking is too flat for that, we don’t think circular. Western, we think too Western.

It can be challenging for clinicians to catch the finer nuances of an interview when they do not understand the language or culture of a patient. Languages also come with specific cultural beliefs:

***P2***: Uhm, so of course culture always is significant. And I think that in a therapeutic role, the patient will at the start already have an idea what sort of gender or age group they would prefer, prefer the, their therapist to be.

Even with the use of an interpreter, these finer nuances can be lost.

Some participants did report that interpreters can help clinicians to understand cultural aspects of the patients’ speech:

***P3***: [Interpreters] help a lot. On a few occasions my interpreter has said, uhm, for example, when we started, uhm, idioms for example is a simple thing. Idioms that we used, the interpreter said no, no, no, no. She realised that they find those idioms difficult to understand. She prefers that we use cultural idioms, and then she used three cultural idioms, and you could immediately see the difference for the patients. The moment you use cultural idioms, you can see the sparkle in their eyes, now they know what we are speaking about. Yes, very interesting.

Still, if a clinician does not have an understanding of a certain culture, information, and thus symptoms, may be misinterpreted. As this participant explained, language and culture can be linked to how individuals use words, and tone. One thus not only needs to understand the language of the patient, but also the culture:

***P5***: … things may be misinterpreted because of culture but, but also misinterpreted because of a lack of understanding of culture …

Some participants also pointed out that it is wrong to assume that just because two people speak the same language, they will share a culture or have the same cultural nuances:

***P3***: You know, it’s very difficult, because culture is not uniform, and I think that is where we sometimes miss it completely. The fact that you are isiXhosa, Afrikaans or English doesn’t necessarily mean that you will have a safari outfit or Brylcream. Do you understand? Some people I would say, even if they are Afrikaans, their culture would fit in more with the English culture, uhm, some of my colleagues who are isiXhosa, are more American than holding onto the isiXhosa culture. So no, you can’t make certain conclusions just because a person is a certain race.

The role of culture and gender can be linked together, as can be seen below. Clinicians need to be sensitive in how they approach interviews when the patient’s gender is different to their own:

***P5***: [Gender] is an issue, I think. I mean, ja. I suppose with that example, talking about sexual side effects, for example, somebody may feel uncomfortable. Uhm, ja. I think I, I was, I’m very aware of it though and try normalise from the beginning and those are things that may be uncomfortable or difficult. I’ve never directly been exposed to someone who’s dismissive of me because of my gender or who’s asked to see a male psychiatrist because of gender. I’ve never had those issues, uhm, personally. But I know, know that it does happen. Both ways, you know. Patients prefer to see somebody of a specific gender sometimes because of previous sexual trauma or previous relationship difficulties. And then I think, ja, culturally, uh uh, some sort of male-dominant culture where the, uhm, the female opinion may not be weighted as strongly. But it, it hasn’t, I think things are changing. I haven’t had dif-, real difficulties.

### The use of trained and untrained interpreters and using staff members as interpreters

As noted, due to the nature of mental health interviewers and the unavailability of interpreters, clinicians are often forced to make use of non-clinical staff at the hospital as ad hoc interpreters. Although staff members are asked to interpret at times, it is important to keep in mind that this is not part of their work descriptions and it can feel quite unfair to ask staff members to act as interpreters:

***P1***: A medical student’s purpose is to learn; a nurse has her own nursing responsibilities. I think it is valuable to have an interpreter that was trained as an interpreter, who knows that it is their role to interpret, instead of doing it ad hoc.

Participants reported that they have made use of security guards, family members, cleaners and anyone available to interpret in the past. One participant reported that they would prefer to make use of clinical staff to help out as an interpreter, but that if such a person is not available, a trained interpreter would be preferable:

***P6***: I prefer clinical staff. Uhm, but if you can’t find clinical staff, then preferably it must be someone that has been trained.

Other participants also reported that, for them, a trained interpreter would be the best option. Even when interpreters are appointed at a hospital, it is important to remember that they might not be available after hours. Due to the nature of the work and patients, clinicians might be forced to ask staff to interpret after hours or when they need an interpreter urgently.

## Discussion

### Difficulties experienced by psychiatrist due to the language barrier

The participants in this study experienced numerous difficulties due to the language barrier. This not only complicates the clinician’s job, but jeopardises the quality of care that patients receive, by no fault of the clinician. It is suggested that important information gets lost during interviews, information which could be vital to the health and care of the patient. This information can get lost without the knowledge of the clinician. The emotional response of patients, an important diagnostic dimension, is also difficult to utilise. Patients might not react as they would when communicating in their first language and clinicians may find it difficult to gauge a patient’s emotional response to questions or the content of what they are discussing.

Patients also tend to revert back to their first language when they are distressed. This can leave clinicians feeling defeated and even frustrated, but it seems unfair to expect a distressed person to communicate in a language that they don’t feel competent in and being forced to communicate in their second or even third language can add to this distress.

The language barrier also puts strain on clinicians’ already full schedules. Not having adequate time to fully assess a patient is not only an issue for the patient where there is a language barrier but may also affect a clinician’s other duties and the time they are able to spend with other patients they still have to see.

A major concern for participants in the present study is that the language barrier affects their ability to form a therapeutic relationship with patients, something which is of vital importance. There is no shortage of evidence on the importance of a strong therapeutic relationship, and the ability to form a strong alliance with patients is one of the cornerstones of ensuring that patients receive good quality health care. Patients are often distressed and in a very vulnerable state, and yet are being asked to be comfortable with a clinician that they cannot communicate with and, possibly, having a third presence in the room. Clinicians are expected to build an alliance with the patient via a third person, which can be a daunting process, and can be compromised by alliances built between the patient and interpreter.

### Difficulties when working with interpreters

Making use of interpreters can counter some of the difficulties experienced due to the language barrier, but this introduces its own issues. Participants in the study noted that information still gets lost when making use of interpreters. There was frustration among participants that interpreters and patients can have a long conversation, but in response the interpreter only conveys a short sentence or phrase to the clinician. One participant seemed to believe that interpreters lead patients into giving certain answers, answers that the interpreters think the patient should give. Due to the nature of interpreting practices in South Africa, where informal interpreters are often the norm, this is a major concern. These ad hoc interpreters are not clinically trained, and this can lead to clinicians receiving incomplete or even completely inaccurate information. Interpreters can also make their own interpretation of what the patient is saying, and as they are not clinically trained, this is a concern. At times interpreters also ‘clean up’ what the patient says, or reports to the clinician that the patient is speaking nonsense. The clinician then does not receive verbatim words and can lose important information, which impacts their ability to do an assessment of the patient.

Confidentiality issues come to the fore, too, when making use of informal interpreters. When making use of security guards, cleaners, family members and sometimes whoever is available to interpret, it becomes difficult to ensure confidentiality. Clinicians must do their best to explain ethical issues to interpreters, but once the interview is over, what the interpreter does with information discussed during the session is out of the clinician’s realm of control.

### The need for language services

Participants in this study noted that there is a need to provide a better service for patients who speak languages other than English and Afrikaans. None of the participants in the study were fluent in any of South Africa’s indigenous languages, with all seven participants only being able to communicate in English and Afrikaans. isiXhosa was the language that was mentioned most often as being encountered by practitioners. Yet, participants reported that if an isiXhosa patient was to come to their ward, they would have a problem. isiXhosa is one of the official languages of the Western Cape, but isiXhosa patients would have great difficulty accessing mental health care services in this area. Further, as pertains to therapy groups, non-English or Afrikaans-speaking patients are unable to participate. Most groups are only conducted in English or Afrikaans, and if a patient is not fluent in these languages, they are unable to join the group. One participant reported that if groups were more language competent, they believe that there would have been more patients that could attend and benefit from the groups that are offered. Patients are thus excluded from a service based on the fact that they are a first language speaker of a language that is one of South Africa’s official languages other than English and Afrikaans, which is a concern.

Finally, clinicians were concerned as they often have to decide on the best possible treatment based solely on how the patient looks and how they behave, the ‘clinical picture’, rather than the clinical interview. This compromises both clinician and patient and can have negative consequences. Participants in the study noted how they are also unable to discern the finer nuances of a patient, which impacts on their ability to make an accurate diagnosis and decide on a treatment plan.

### Participants’ views on interpreters

The majority of the participants in the present study reported that interpreters can be an effective and useful resource, if used correctly. Previously, interpreters had been appointed at the hospital where the study was conducted, but this service has since stopped. Even though this service was available, it was not always viable to make use of the interpreters; as one participant noted, they saw the interpreters, possibly, once, but never made use of their services. Participants noted that it was difficult to make contact with the interpreters and to make appointments with them. One of the benefits of having formal interpreters appointed, was that groups were able to be conducted in a language other than English and Afrikaans. During this period, isiXhosa groups were facilitated. An important service was thus available to a wider population.

### The role of age and gender

Participants reported that patient age had the most apparent impact when interviewing an elderly patient. When working with an elderly patient, clinicians are expected to show a certain level of respect. A patient’s age might, however, also be linked to their competency in a certain language. One participant reported that, based on statistics, older patients that are first language isiXhosa speakers might have a lower level of education due to South Africa’s past. As a result, these patients might be less able to communicate in English than the younger generation.

The role of gender was mostly considered in its relation to the patient’s culture, as there can be a strong link between the two. Different cultures have different gender roles, and clinicians have to be sensitive and respectful of this. When interviewing distressed patients who were the victim of gender violence, or abused, clinicians have to be particularly sensitive. There is a certain dynamic in the room when clinician and patient are different genders, and clinicians have to be sensitive to the patient’s reaction towards them.

### The role of culture

In the present study, culture was found to have a profound impact. Often when there was a language barrier, patient and clinician were also from different cultures. Even when patient and clinician can communicate, their way of using a language might differ. Some cultures make use of a more symbolic way of communicating. In South Africa, the psychiatric paradigm is largely Western. Working from this Western frame, and lacking knowledge about the patient’s culture, can lead to miscommunication and misunderstandings, with the result that clinicians might make errors in diagnosis or treatment. Specific cultures might also have their own nuances, which can be missed or interpreted differently. Interpreters can play a role here, in helping clinicians understand the culture of a patient. However, it also should not be assumed that people that share a language will also share a culture. This complicates the idea of interpreters acting as cultural brokers.

### Trained interpreters or staff members

Four of the seven participants reported that for them, having access to a professional interpreter would be the best solution to overcoming the language barrier. Other participants also reported advantages of having professional interpreters but noted that this is not always a viable option. These interpreters might not be available after hours, and at times the clinicians need to make a decision immediately and can not necessarily wait for an interpreter.

Three participants seemed to prefer making use of staff members, but only one participant stated outright that they would prefer to make use of clinical staff to help out as an interpreter. Making use of clinical staff can be valuable, as they are clinically trained and have a better understanding of the psychiatric process (although it may create role conflict). Although the other two participants did not directly say that they prefer staff members, they indicated that this is usually their only option and that they have made use of staff members as interpreters on a regular basis, whether this be security guards, cleaners or any available colleague. There are, however, numerous difficulties with this, some of which have already been highlighted in this paper. Other staff members are employed to have another function, not act as interpreters, so it can be very unfair to expect them to carry out a task that is not part of their work description. Although participants had their preferences as to who they would like to have as interpreter, the reality remains that they usually have to settle for whoever is available.

### Understanding the data in context

This study was limited in scope and served mainly to elicit the views of people providing services in a complex mental health system in a divided society. Between the lines, we can see evidence of clinicians who want to do their best, but who feel inadequate to the complexity of their role in a multilingual context in a highly divided society. This is a crucial issue to consider in any future interventions. It is important to note that the participants are inserted into a broader system over which they have very little control, but which through their professional work they play a role in reproducing. For example, though clinicians express a need for improved and expanded interpreter services, as we have shown in other research [3], the bureaucratic function of the institution, paradoxically, pulls the few resources that are available away from improving clinical care towards serving administrative functions. Managers of personnel who provide interpreting services in this context do not work in clinical roles. The need to perform administrative tasks is a key feature of all bureaucracies, but where resources are scarce, the administrative load may detract from the reaching of clinical ends.

It is also important to acknowledge that in this context, as in others, the role and function of psychiatry itself is not just patient care but also working through the load of a large number of patients – in this context clinicians may feel pressured to complete tasks and to become ‘irritated’, as one of our participants put it, when language barriers slow things down. It would be all too easy to blame clinicians for not being the best clinicians they can be in a context like this, but as we have suggested elsewhere [4] and as other work in similar hospital settings in South Africa also suggests [51], it is more helpful to try to understand contexts and to change them than to blame front-line personnel. In this regard, it is important to work towards improved skilling of psychiatrists and similar personnel in how to work with interpreters, as we have discussed in other work [3,48].

## Conclusion

This study has important limitations. It was limited in scope, as not all issues pertaining to the language barrier could be addressed. One important limitation is that the study was conducted in only one psychiatric hospital, so we could only hear from psychiatrists working in one of many catchment areas in the Western Cape. Data were thus limited to a very specific population. Studies conducted in different catchment areas will likely raise different issues. Despite these limitations, the findings have important implications.

The most important finding is that psychiatrists experience numerous difficulties in conducting their work due to the language barrier. This has an impact on their ability to provide adequate mental health care to patients. There is a need for better language services to ensure that everyone that seeks mental health care can receive the same level of care. These services require skilled interpreters and clinicians skilled to work with interpreters. Currently, one’s ability to speak a certain language will have a significant impact on the quality of care received. Ultimately, it is the right of the patient to communicate in their home language, especially where that language is an official language. Making use of make-shift plans, like ad hoc interpreters, at least offers these people access to the health care system. This is, however, not a very viable or long-term option.

## Paper context

In South African mental health care, people who do not speak certain languages often receive treatment from a psychiatrist with the assistance of an untrained interpreter. Our qualitative study shows that South African psychiatrists experience numerous difficulties in conducting their work due to the language barrier, and this diminishes the quality of care patients receive. There is a need for better language services to ensure that everyone who seeks mental health care receives optimal care.
